# Guidelines for reproducible analysis of adaptive immune receptor repertoire sequencing data

**DOI:** 10.1093/bib/bbae221

**Published:** 2024-05-15

**Authors:** Ayelet Peres, Vered Klein, Boaz Frankel, William Lees, Pazit Polak, Mark Meehan, Artur Rocha, João Correia Lopes, Gur Yaari

**Affiliations:** Faculty of Engineering, Bar Ilan University, 5290002 Ramat Gan, Israel; Bar Ilan institute of nanotechnology and advanced materials, Bar Ilan university, 5290002 Ramat Gan, Israel; Faculty of Engineering, Bar Ilan University, 5290002 Ramat Gan, Israel; Bar Ilan institute of nanotechnology and advanced materials, Bar Ilan university, 5290002 Ramat Gan, Israel; Faculty of Engineering, Bar Ilan University, 5290002 Ramat Gan, Israel; Bar Ilan institute of nanotechnology and advanced materials, Bar Ilan university, 5290002 Ramat Gan, Israel; Institute of Structural and Molecular Biology, Birkbeck College, University of London, London, United Kingdom; INESC TEC – Institute for Systems and Computer Engineering, Technology and Science Porto, Portugal; Faculty of Engineering, Bar Ilan University, 5290002 Ramat Gan, Israel; Bar Ilan institute of nanotechnology and advanced materials, Bar Ilan university, 5290002 Ramat Gan, Israel; INESC TEC – Institute for Systems and Computer Engineering, Technology and Science Porto, Portugal; INESC TEC – Institute for Systems and Computer Engineering, Technology and Science Porto, Portugal; INESC TEC – Institute for Systems and Computer Engineering, Technology and Science Porto, Portugal; Faculty of Engineering, Bar Ilan University, 5290002 Ramat Gan, Israel; Bar Ilan institute of nanotechnology and advanced materials, Bar Ilan university, 5290002 Ramat Gan, Israel

**Keywords:** AIRR-seq, reproducibility, FAIR, preprocessing, annotation, pipelines

## Abstract

Enhancing the reproducibility and comprehension of adaptive immune receptor repertoire sequencing (AIRR-seq) data analysis is critical for scientific progress. This study presents guidelines for reproducible AIRR-seq data analysis, and a collection of ready-to-use pipelines with comprehensive documentation. To this end, ten common pipelines were implemented using ViaFoundry, a user-friendly interface for pipeline management and automation. This is accompanied by versioned containers, documentation and archiving capabilities. The automation of pre-processing analysis steps and the ability to modify pipeline parameters according to specific research needs are emphasized. AIRR-seq data analysis is highly sensitive to varying parameters and setups; using the guidelines presented here, the ability to reproduce previously published results is demonstrated. This work promotes transparency, reproducibility, and collaboration in AIRR-seq data analysis, serving as a model for handling and documenting bioinformatics pipelines in other research domains.

## INTRODUCTION

In today’s research landscape, there is a noticeable shift toward prioritizing the findability, accessibility, interoperability, and reusability (FAIR) of research data and processing pipelines, as highlighted by the FAIR principles [1]. These principles serve as a framework to enhance the overall quality and impact of scientific endeavors. FAIR is commonly discussed in terms of data sharing, however, it can also refer to analysis workflows. In this context, other than FAIRness, data science places special emphasis on the reproducibility of pipelines [2, 3]. Reproducibility refers to the ability to design and implement data analysis procedures in a manner that allows the obtained results to be faithfully replicated in the future by independent researchers. This ability is critical to scientific progress, as it allows researchers to build upon previous findings, and ensure that research findings are reliable and can easily be validated by others.

Several tools offer workflow management systems that are designed to facilitate the development and execution of computational workflows in a reproducible and consistent manner. Each system has its own strengths and weaknesses, and the choice of which system to use often depends on the specific requirements of the project, the expertise of the user, and the availability of computational resources [4]. Snakemake [5], for example, is a Python-based system that is well-suited for small-to-medium-sized workflows and has a user-friendly interface, while Cromwell [6] is a highly scalable system that is designed to run on cloud computing resources and supports multiple workflow languages. Galaxy [7] is a web-based platform that provides a graphical user interface for the creation and execution of workflows, while Toil [8] is a Python-based system that provides low-level control over workflow execution and supports distributed computing. Nextflow [9] is a highly portable and scalable system that supports a wide range of computing environments, and ViaFoundry (a reimagination of DolphinNext) [10] is a user-friendly platform that provides a well-architected user interface for the creation and execution of bioinformatics workflows. Yet, despite the existence of these systems, common computational pipelines still face many challenges, such as data privacy, replication of the execution environment, and availability of adequate computational resources [11, 12]. Therefore, researchers must carefully evaluate the strengths and limitations of different systems and select the most appropriate one based on their specific needs and circumstances to maximize the reproducibility of their work.

Adaptive Immune Receptor Repertoire sequencing (AIRR-seq) data can be used to characterize the diversity and specificity of T and B cell receptors in the immune system [13]. AIRR-seq data are generated using high-throughput sequencing of the immune receptors. These data store critical information on the immune response to infections, diseases, and vaccines. The analysis of AIRR-seq data involves a series of computational and statistical methods [14], including quality control [15–17], read alignment [18, 19], sequence clustering [20–22], annotation [23–26], and comparative analysis [27–29].

AIRR-seq data is highly diverse in terms of production (e.g. sequencing platforms and library preparation protocols) and processing tools [30]. This has led to a broad range of analysis pipelines, and non-standard reporting on the steps, tools, and parameters used, posing a challenge for reproducing results from published datasets. Therefore, the FAIR principles and reproducibility are particularly important, for enabling efficient sharing and integration of AIRR-seq data across research groups and projects [31].

Several FAIR challenges can arise when creating and using analysis pipelines, such as variability in tools and programming languages, complexity of pipeline creation and parameters, lack of documentation, difficulties in sharing pipelines, hardware and software dependencies, and human error. Considering these challenges, we have derived a set of guidelines for creating, tweaking, and running analysis pipelines. The guidelines, presented here for AIRR-seq analysis, have been formulated using ViaFoundry [10] coupled with tools for controlling the run environment, pipeline documentation, and archiving. These guidelines ensure the reproducibility and adherence to FAIR principles in AIRR-seq data analysis pipelines.

By following these guidelines and leveraging ViaFoundry’s capabilities for pipeline creation, parameter configuration, environment management, and documentation, researchers can ensure that their AIRR-seq analysis pipelines are both reproducible and aligned with FAIR principles. This comprehensive approach not only facilitates transparent research but also promotes collaboration and knowledge dissemination within the scientific community.

## RESULTS

### Implementation of published pipelines

Despite community efforts in setting standards for documenting AIRR-seq data production and associated metadata [30], the analysis steps are still reported in an unstructured text format. This often results in inaccuracies of crucial details that are imperative for reproducing the analysis. To address this issue, we compiled ready to use analysis pipelines in a format that stores sufficient information of the analyses. We have collected from the literature several promising pipelines that are used in many AIRR-seq studies. The identified pipelines constitute the foundation for the compiled list. Our intention is that when new protocols or analyses emerge, the established pipelines can be easily adapted.

We suggest an approach that simplifies complex pipelines and ensures their reproducibility. The approach relies on a combination of a scripting language, versioned containers, documentation and archiving. Specifically, we use ViaFoundry [10], which is a user interface wrapper to the scripting language Nextflow [9]; Docker [32] or Singularity [33] images as containers; Git [34] for documentation; and Zenodo [35] for archiving, which ensures long-term accessibility and traceability.

The ten common pipelines presented in this study are listed in Table 1. These pipelines, along with their associated Zenodo DOIs and ViaFoundry pipeline number, are available on our GitHub page (https://github.com/PipeAIRR). Researchers can freely explore the various options and select the pipeline that best fits their needs, as explained in the next section.

**Table 1:** AIRR-seq pipelines implemented

**Pipeline**	**Input data**	**Sequencing protocol**	**UMI**	**Published paper(s)**	**GitHub Archive**	**Zenodo DOI**	**Via-Foundry**
RP1	Raw	2X250	+	[36]	PipeAIRR/RP1	10783397	381
	sequences						
RP2	Raw	2X250	–	[37]	PipeAIRR/RP2	10783402	382
	sequences						
RP3	Raw	5’ RACE	+	[38, 39]	PipeAIRR/RP3	10783403	383
	sequences	325+275					
RP4	Raw	2X300	+	[40]	PipeAIRR/RP4	10783401	386
	sequences						
RP5	Raw	5’ RACE	+	[41]	PipeAIRR/RP5A	10783404,	390,
	sequences	325+275			PipeAIRR/RP5B	10783407	393
RP6	Raw	Roche 454	–	[42]	PipeAIRR/RP6	10783408	396
	sequences	BCR mRNA					
RP7	Raw	2X125 CD4	–	[43]	PipeAIRR/RP7	10783409	397
	sequences	T cells RNA					
PP1	Processed sequences	–	–	[44]	PipeAIRR/PP1	10783412	401
PP2	Processed sequences	–	–	[27]	PipeAIRR/PP2	10783413	402
PP3	Processed sequences	–	–	[26]	PipeAIRR/PP3	10783414	398

### Tweak and run pipelines

**Figure 1:**
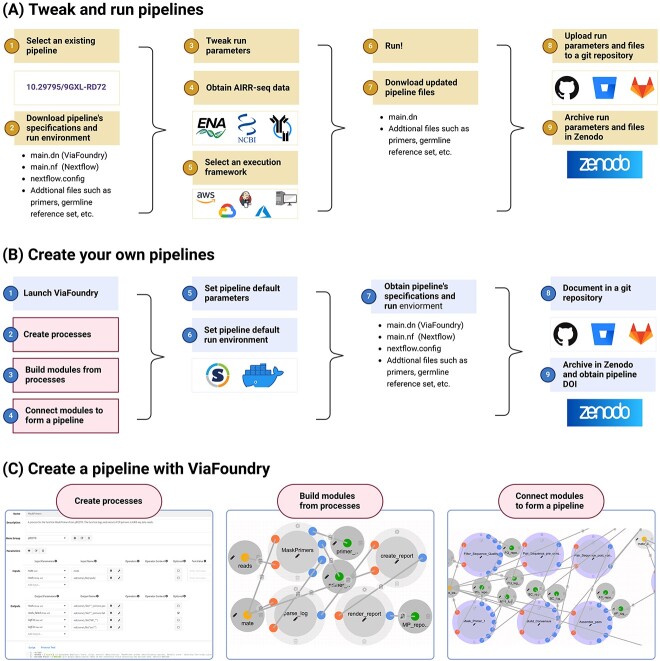
Steps for reproducible AIRR-seq analysis pipelines. (**A**) Tweak and run existing pipelines. In step one, an existing pipeline is selected using its Digital Object Identifier (DOI). In step two, the pipeline’s specification and run environment files are downloaded. In step three, the run parameters (e.g., process parameters, primer files, etc.) are adjusted. In step four, AIRR-seq data is obtained from public databases (e.g., ENA, NCBI) or from local storage. In step five, the execution framework is selected, which can be cloud-based (e.g., AWS, Azure, Google) or using ViaFoundry execution framework server or locally run in an automation server platform management (e.g., Jenkins). In step six, the analysis is run in the selected framework. Lastly, the updated pipeline files are downloaded in step seven and documented and archived for future use in steps eight and nine. (**B**) Create and archive pipelines. In steps one to six, the ViaFoundry framework is used to create the analysis pipeline and set the parameters and run environment. In step seven, the pipeline specification and run environment are obtained. Lastly, the files are documented in a Git repository and archived in Zenodo in steps eight and nine. (**C**) Create a pipeline with ViaFoundry. The first step is creating processes using the dedicated GUI. The second step is combining different processes into a module. The third step is assembling the full pipeline for analyzing AIRR sequences from a set of modules. This figure was created with BioRender.com

The recommended workflow for reproducible analysis of AIRR-seq data is shown in Figure 1A. To begin, users should identify the pipeline that best aligns with their analysis protocol. As mentioned earlier, we have created a resource where pipelines are available for selection. To access the pipeline, users should download the corresponding version from Zenodo, which includes three main files: a ViaFoundry pipeline file (main.dn), a Nextflow pipeline file (main.nf), and a Nextflow configuration file (nextflow.config). Additionally, the archive may contain other essential files needed for running the pipeline, such as a primer sequence file or a germline reference set file. Once users downloaded the pipeline, they can inspect and tweak its parameters. This can be done by using ViaFoundry and uploading the main.dn file. By loading the file into the ViaFoundry, the users can see the pipeline and have access to all the processes and modules that were defined. Further, in the Advanced tab of the pipeline screen (Supplementary Figure 4) the user can find the pipeline fixated parameters as well the additional files (Supplementary Figure 6). AIRR-seq data for the analysis pipeline can be obtained either from locally available files, or from public databases like ENA [45], NCBI [46], iReceptor [47], and OAS [48]. Then, the execution framework for the analysis is selected. The easiest option is to use ViaFoundry’s own execution platform. Alternatively, the Nextflow main.nf file can run either on a local machine, or on a cloud server such as AWS, Google Cloud, and Azure. Additionally, Jenkins can be employed for effective server management, again either on local or cloud machines. After running the pipeline with the potentially modified parameters and run environment, the updated pipeline specification files need to be downloaded from a ViaFoundry instance, including the tweaked main.dn and nextflow.config files, and any additional files required to run the analysis. These files should be carefully documented in a Git archive, following a concise template that outlines the utilized pipelines, modified parameters, a brief description of the primers or germline set, and any other relevant details. To ensure long-term preservation and accessibility, this Git repository should be archived in Zenodo, resulting in an allocation of a unique DOI. The obtained DOI can be used to reproduce the results, and should be clearly mentioned in any related publication.

### How to create your own reproducible pipeline

For researchers with specific requirements not addressed by the existing pipelines, it is also possible to create a *de novo* pipeline. The creation of reproducible and coherent pipelines can be challenging due to variations in tools and programming languages. Figure 1B illustrates the necessary steps to establish a reproducible pipeline. Steps one to four describe the basic creation of analysis pipelines using ViaFoundry. Initially, the users generates a set of processes, where each process represents an operation that can be performed on the AIRR-seq data or serves as a prerequisite for downstream analysis (Figure 1C, left). Subsequently, modules are formed by linking processes that are intended to work together as a cohesive unit (Figure 1C, middle). Users can also reuse existing modules from other pipelines, by uploading the main.dn file that is embedded in their ViaFoundry instance. Finally, the modules are interconnected to form the final analysis pipeline using a straightforward drag and drop approach (Figure 1C, right). ViaFoundry further allows controlling the version of each of the processes, modules, and pipelines. Steps five and six in Figure 1B refer to setting the pipeline’s parameters and run environment in the Pipeline Header Script section. Both the parameters and the run environment are set using the Groovy programming language, where the environment is defined by either a versioned Docker or Singularity image that the pipeline executes. These images can be built or retrieved from online resources such as DockerHub (http://dockerhub.com/) or Singularity Hub (https://singularityhub.github.io/). Subsequently, the pipeline can be executed using ViaFoundry to obtain the desired products. In step seven, the user should obtain the pipeline’s specifications and run environment that they have defined in previous steps. This includes the pipeline’s ViaFoundry file (main.dn), Nextflow script, configuration files (main.nf and nextflow.config), and any additional files. On top of the obtained information from step seven, step eight adds a detailed documentation of the pipeline specifications in a Git repository. In step nine, the repository is archived in Zenodo, and a DOI for the archived version is obtained. This DOI can then be utilized to cite the pipeline in future works and reproduce the results.

### A case study: reproducing published results

**Figure 2:**
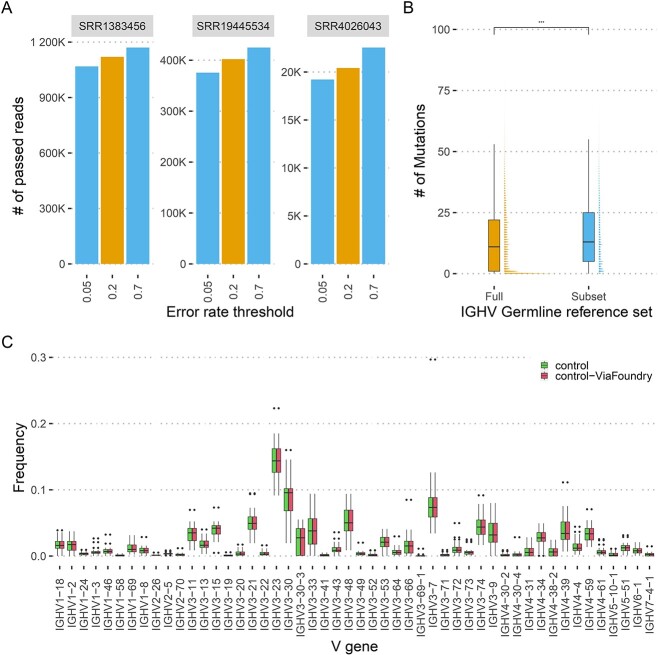
A case study of reproducing AIRR-seq analysis results. (**A**) The influence of a single pipeline parameter on the number of passed reads. Each facet is an independent repertoire, the x-axis corresponds to different error rate thresholds used in the MaskPrimers process, and the y-axis is the number of reads that passed the process given the threshold. Yellow bars correspond to the original threshold used to analyze the repertoires, and blue bars correspond to the alternative thresholds (**B**) The influence of initial IGHV germline reference set on mutation load. The x-axis corresponds to the different IGHV germline reference set. The yaxis corresponds to the calculated mutation load. (**C**) IGHV gene mean usage. The x-axis corresponds to the different IGHV genes, and the y-axis corresponds to the mean usage frequency across all control individuals. Green boxes represent the original publication results, and red boxes represent the results obtained by pipeline PP1 listed in Table 1.

Reproducing AIRR-seq data analysis is an error-prone process, as highlighted earlier. Misinterpretation of any specification can significantly impact the final outcome. Here, we showcase the effect of two tuneable parameters that significantly alter the final outcome. Specifically, we demonstrate that the number of quality filtered reads is not meaningful unless the process and parameters are understood. We explored the influence of the error rate threshold of the MaskPrimers process, a common step across many AIRR-seq analysis pipelines to identify primer sequences in the data. An error rate is calculated for each sequence, indicating the lowest percentage of mismatches from one of the primer sequences. The pass or fail status of each sequence is determined using an error rate threshold. To analyze the effect of different thresholds, we applied three thresholds of the MaskPrimers process to three independent repertoires (Figure 2A). As expected, alteration of the thresholds resulted in significant differences in the number of sequences passing the process. We next explored the impact of the germline reference set on annotating AIRR-seq data. After processing the raw AIRR-seq reads, the subsequent step involves annotating the sequences with their respective V(D)J allele assignments. One common approach is to utilize a germline reference set of V(D)J alleles [49] and an alignment software such as IgBlast [18]. To assess the effect of the germline reference set, we annotated 30 individuals repertoire from PRJEB28370 using both a immunoglobulin heavy chain V (IGHV) allele germline reference set downloaded from IMGT (July 2022) and a filtered set that includes a single allele per gene. Comparing the calculated mutation load of the sequences annotated using the full set with the mutation load calculated from the filtered set yields a significant increase in the mutation distribution of the latter (Figure 2B). These two examples highlight the importance of accurately reporting all steps and parameters that were used for the analysis.

Finally, we aimed to replicate an analysis from a recent publication, which includes an AIRR-seq dataset of human heavy chain repertoires [44]. This publication explored differences between individuals with Crohn’s disease and control subjects. Specifically, in Figure 2D of that publication, the authors presented the variation in the mean usage of the top 50 IGHV genes. The Methods section of the publication describes the steps for the analysis, and the archived code to produce the figure is available on GitHub https://github.com/saframodi/crohnData. Utilizing the publicly available AIRR-seq data from the above mentioned paper (NCBI BioProject accession number: PRJNA788351), we applied the PP1 ViaFoundry pipeline listed in Table 1 exclusively to the control cases from the paper. Figure 2C illustrates a successful replication, perfectly matching the published results.

## DISCUSSION

In this study, we underscore the importance of clear, detailed documentation and the creation of a community repository to enhance the comprehension and reproducibility of AIRR-seq data analysis. We implemented ten common pipelines using ViaFoundry, a user-friendly interface for the Nextflow scripting language, and employed Docker and Singularity images as versioned containers, Git for documentation, and Zenodo for archiving. The resource presented here offers a collection of ready-to-use pipelines with accompanying documentation, making the process of AIRR-seq data analysis more transparent and less error-prone. We demonstrated the application of the approach and its implementation, highlighting the sensitivity of AIRR-seq data analysis to parameters and setups. Furthermore, we showcased the ability of the suggested approach to efficiently reproduce results from previously published works.

We addressed several challenges in building or utilizing AIRR-seq analysis pipelines, such as the lack of structured documentation, inconsistencies in dataset versions across public databases, and the need for archiving accompanying files. To overcome these, we introduced a structured documentation repository, specified dataset versions, and offered guidelines for parameter customization. We also emphasized the importance of automating the pre-processing analysis steps to ensure standardized execution, reduce human error, and enable the creation of shareable and reproducible pipelines. The challenges and solutions presented above are summarized in Table 2.

**Table 2:** Challenges in creating reproducible pipelines and their respective solutions

**Challenges**	**Solution**
Variability in tools and programming languages	Use a workflow management system that supports a variety of tools and programming languages, such as ViaFoundry.
Complexity of pipeline creation	A step-by-step guide to creating a reproducible pipeline, using a platform like ViaFoundry is provided.
Lack of parameters documentation	Configure and set the pipelines parameters and run environment with ViaFoundry to create a uniform documentation, that can be easily used.
Difficulties in sharing pipelines	Archive the pipeline in a repository like Zenodo.
Hardware and software dependencies	Configure and set the pipeline’s parameters and runtime environment using ViaFoundry to generate uniform documentation that is easily accessible.
Human error	Implement quality control measures by adding reports that can be displayed in ViaFoundry.

The ability to modify pipeline parameters according to specific research needs is a critical feature. We provided a framework for documenting changes made in the analysis process, using Git repositories and Zenodo archiving. Furthermore, we explain how to create *de novo* pipelines that are both reproducible and comprehensible, using ViaFoundry’s interface and Docker and Singularity images.

Peer reviewers play a critical role in validating the findings and ensuring the scientific rigor of a study prior to publication. By providing detailed documentation, versioned pipelines, and archived files, the presented approach will enable reviewers to easily replicate analyses and verify the reported results.

We have thoroughly documented and stored our executed pipeline on the following GitHub repository: https://github.com/PipeAIRR. Additionally, we have formulated a complementary tutorial guide available at https://pipeairr.github.io/pipeAIRR/.

Overall, our work offers a valuable resource for AIRR-seq data analysis, promoting transparency, reproducibility, and collaboration within the scientific community. The approach outlined here ensures adherence to the FAIR principles and promotes reproducibility through the following means:

- **Findable:** The complete ViaFoundry pipeline is stored within a Git repository, providing a searchable and locatable resource.
- **Accessible:** The Zenodo archive DOI ensures long-term access to the cited pipeline, making it readily available to the research community.
- **Interoperable:** Building pipelines through ViaFoundry’s graphical user interface simplifies the understanding of the pipeline’s steps and parameters, enhancing interoperability.
- **Reusable:** The use of Docker or Singularity images guarantees that the execution environment is reusable on various platforms, facilitating the execution of the pipeline across different systems.
- **Reproducibility:** Adhering to the guidelines for pipeline creation, parameter configuration, and documentation within ViaFoundry, along with citing the pipeline’s Zenodo DOI, ensures the reproducibility of research results.

As the field of AIRR-seq data analysis continues to evolve, we anticipate the repository presented here to be an integral part of the process, encouraging the development of more efficient, adaptable, and reproducible pipelines. We believe that our approach can serve as a model for handling and documenting bioinformatics pipelines in other research domains as well.

## METHODS

### Creation of reproducible pipelines with ViaFoundry

A detailed explanation for creating reproducible pipelines with ViaFoundry can be found in the Supplementary Methods section. Briefly, ViaFoundry pipelines are constructed using two fundamental components: processes and modules. These building blocks are seamlessly assembled using a drag-and-drop approach to create complete, customized pipelines. Users have the flexibility to tailor these pipelines to their specific needs, configuring parameters, and defining the execution environment through Docker or Singularity containers. For further documentation, customization, and collaborative development, ViaFoundry pipelines can be efficiently exported and integrated into a Git repository.

### Creation of a run execution environment with Docker or Singularity

The Docker and Singularity containerization tools allow encapsulating an entire execution environment, including the operating system, libraries, dependencies, and software, into a self-contained image that can be easily shared and executed on various systems. The Docker or Singularity images should be well documented and accessible through either DockerHub (http://dockerhub.com/) or Singularity Hub (https://singularityhub.github.io/).

### Documentation and archiving with Git and Zenodo

We employed Git and Zenodo to systematically document and archive our ViaFoundry pipeline. ViaFoundry facilitated comprehensive documentation by allowing us to directly integrate the pipeline, configuration file, and additional files into a Git repository. This detailed description within ViaFoundry automatically translated into a well-structured README.md file within the Git repository, ensuring the preservation and accessibility of the pipeline. For robust archiving, we harnessed the capabilities of Zenodo. The Git repository, containing our meticulously documented pipeline, was uploaded to Zenodo. By connecting the Git repository with Zenodo we can automaticly generate a DOI that will update with any update to the repository, guaranteeing the citability of the research work.

**Key Points**

- We illustrate the significance of adhering to FAIR principles (findability, accessibility, interoperability, and reusability) and ensuring reproducibility in analysis pipelines.
- We outline the challenges encountered in AIRR-seq data analysis, such as tool variability, pipeline complexity, and insufficient documentation.
- Underscoring the importance of transparent documentation, versioned pipelines, and archived files for peer reviewers, we provide a GitHub repository and a tutorial to support their work.
- Our guidelines for crafting reproducible AIRR-seq analysis pipelines address challenges like tool variability, complexity, documentation gaps, sharing obstacles, dependencies, and human error.
- We created a repository which holds 10 common analysis pipelines, and added a complimentary site with guidance. The pipelines can be easily retrieved, used, and later on cited as necessary.

## Supplementary Material

supplementary_bbae221

## Data Availability

The data for Figure 2 are available in Zenodo https://doi.org/10.5281/zenodo.8346877

## CODE AVAILABILITY

All the pipelines are available in the GitHub repositories https://github.com/PipeAIRR
