# Supplementary material for: Guidelines for reproducible analysis of adaptive immune receptor repertoire sequencing data
Source: Brief Bioinform. 2024 May 15;25(3):bbae221. doi: 10.1093/bib/bbae221 (PMC11097599; doi:10.1093/bib/bbae221)
Supplement: supplementary_bbae221 [file supplementary_bbae221.pdf]

Guidelines for Reproducible Analysis of Adaptive Immune Receptor Repertoire Sequencing Data

Ayelet Peres[1,2], Vered Klein[1,2], Boaz Frankel[1,2], William Lees[3], Pazit Polak[1,2], Mark Meehan[4], Artur Rocha[4], João Correia Lopes[4] and Gur Yaari[1,2,†]

[1]Faculty of Engineering, Bar Ilan University, 5290002 Ramat Gan, Israel
[2]Bar Ilan institute of nanotechnology and advanced materials, Bar Ilan university, 5290002 Ramat Gan, Israel
[4]INESC TEC Institute for Systems and Computer Engineering, Technology and Science Porto, Portugal
[3]Institute of Structural and Molecular Biology, Birkbeck College, University of London, London, United Kingdom
[†]To whom correspondence should be addressed. Email: gur.yaari@biu.ac.il

April 22, 2024

Extended methods

ViaFoundry pipeline creation

Create processes

In ViaFoundry, the core element is a process that can deploy operations on different kinds of data. The creation of a process is divided into two sections. The first defines the inputs and outputs of the operation, and the second includes the script to execute and editable default parameters. Supplementary Figure 1 shows an example of a ViaFoundry process for the pRESTO [1] function MaskPrimers, which tags the raw sequences of AIRR-seq data based on a given primer set. The process must receive either one or two raw AIRR-seq files and a primer set file, and returns the tagged reads and the function log file. For this function, a set of optional parameters was defined as well, with the default values suggested by the function. If untouched, the function uses the default values.

Build modules from processes

A ViaFoundry modules is a set of connected processes that should work as one unit. This means that in the complete pipeline, the processes in this unit should not be broken into subunits. For example, Supplementary Figure 2 shows the module MaskPrimers from pRESTO [1]. In this module, we define two nodes, one for tagging the sequences using the MaskPrimers process, and a second node for processing the outputted log files. Though this is a simple example, modules can be more complex with many more processes that work consecutively, in which the complete unit is modular but the steps inside it are fixed.

Connect modules to form a pipeline

Upon creating a bank of processes and modules, we can proceed to assemble a ViaFoundry pipeline. Assembly of a pipeline (Supplementary Figure 3) requires a simple drag and drop method to connect the input nodes (depicted as yellow nodes) and the desired processing modules (depicted as purple nodes). More processing modules can be added and connected to their respective outputs and inputs, as needed.

Set pipeline default parameters and run environment

The parameters and run environment for each pipeline are written using the Groovy programming language. The configuration file (`nextflow.config`) is found at the advance tab of ViaFoundry pipeline configuration window (Supplementary Figure 4). To set the parameters of a process, the user must first access the `params` variable, then the module name, the process name, and finally the parameter name. Run environment can also be set in the configuration file, as can be seen in lines 28 to 35 in Supplementary Figure 4. Different run environments can be set for various machines, as can be seen in the `if and else` statement therein. The `$HOSTNAME` variable holds the machine name, where `default` is usually the local machine. To control the Docker or Singularity image, the `$DOCKER_IMAGE` variable or the `$SINGULARITY_IMAGE` variable needs to be allocated, as in line 29. Moreover, line 32 illustrates the ability to define Docker or Singularity properties. Additionally, lines 33 and 34 demonstrate the configuration of machine properties specifically for remote machines. An important step to ensure the image and parameters are initiated upon running the pipeline using ViaFoundry or Nextflow, is to encapsulate the scripts with the `//* autofill` statement, as shown in lines 27 and 36 (Supplementary Figure 4).

Exporting a ViaFoundry pipeline

Once the pipeline has been assembled and the parameters have been defined, along with any additional files added to the pipeline's environment (Supplementary Figure 6), it can be exported. The exported pipeline consists of several files. The `main.dn` file is an encrypted file specific to ViaFoundry. This file contains crucial information about the pipeline, including the processes and modules used, the pipeline configuration from both the `Pipeline Header Script` section, the `nextflow.config` file, and any additional files integrated into the environment. To access and utilize this file, it can only be opened within an instance of the ViaFoundry server. Once loaded, the user gains access to all the components of the pipeline, allowing them to examine each process and module separately, thereby facilitating the creation of new pipelines based on existing ones. The second file in the exported pipeline is `main.nf`, which is a plain Nextflow script file that can be executed using the Nextflow system. The third file, named `nextflow.config`, incorporates both the configuration made in the `Pipeline Header Script` section and any additional configuration written directly into the file. Lastly, the exported pipeline includes any additional files that were added by the user, such as primer, reference set files, or configuration files.

References

[1] Jason A. Vander Heiden, Gur Yaari, Mohamed Uduman, Joel N.H. Stern, Kevin C. O'Connor, David A. Hafler, Francois Vigneault, and Steven H. Kleinstein. presto: a toolkit for processing high-throughput sequencing raw reads of lymphocyte receptor repertoires. *Bioinformatics*, 30(13):1930–1932, 2014.

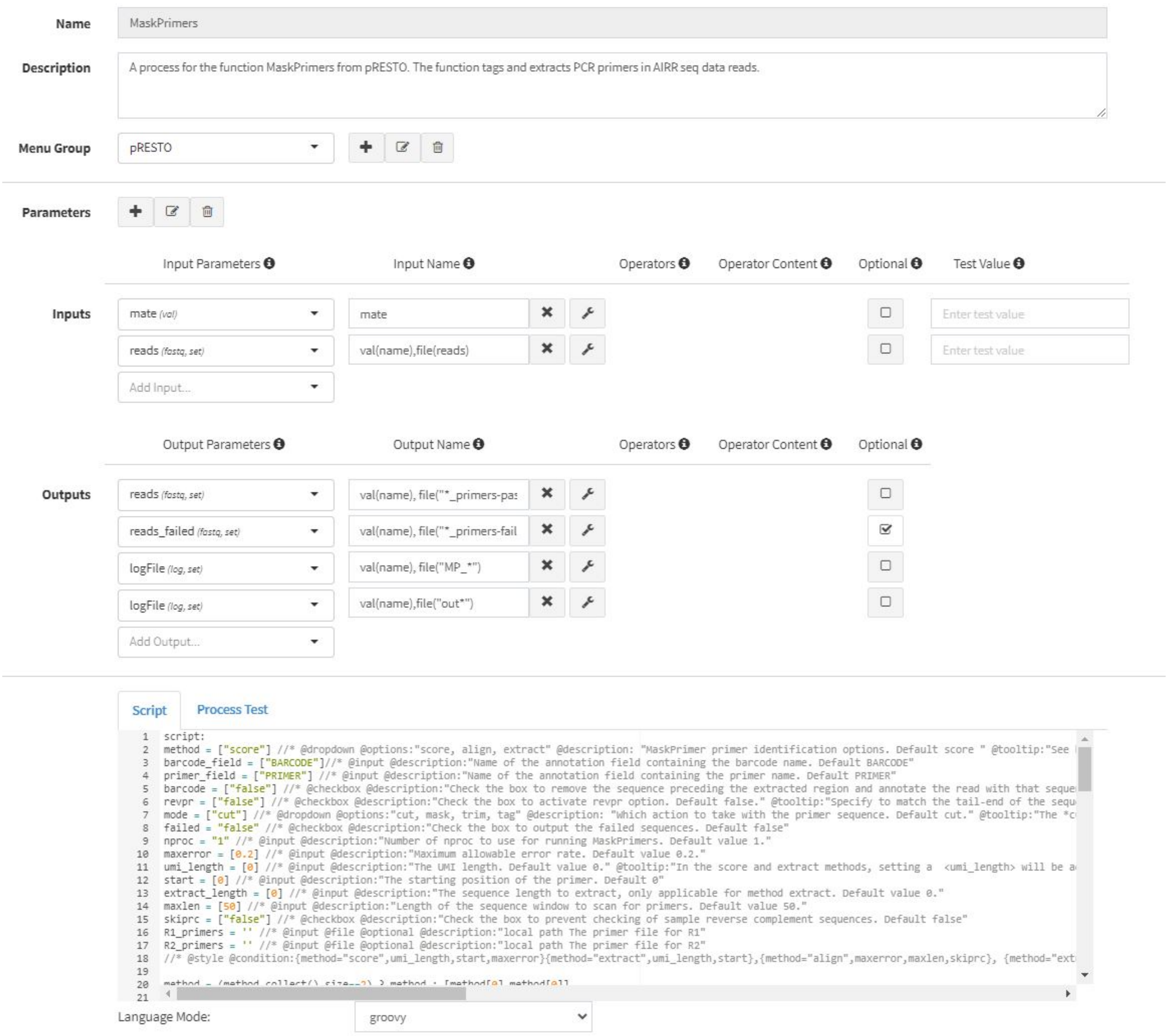

Figure S1: **ViaFoundry MaskPrimers process.** A screenshot of the ViaFoundry process creation page. The page is divided into three sections. The first, defines the name of the process, the description, and the group allocation within the ViaFoundry interface. The second section, defines the required and optional parameters, and the process output. The last section, includes the executed script and the editable default parameters. The script can be written in multiple programming languages such as Groovy, shell, Perl, R, python, etc.

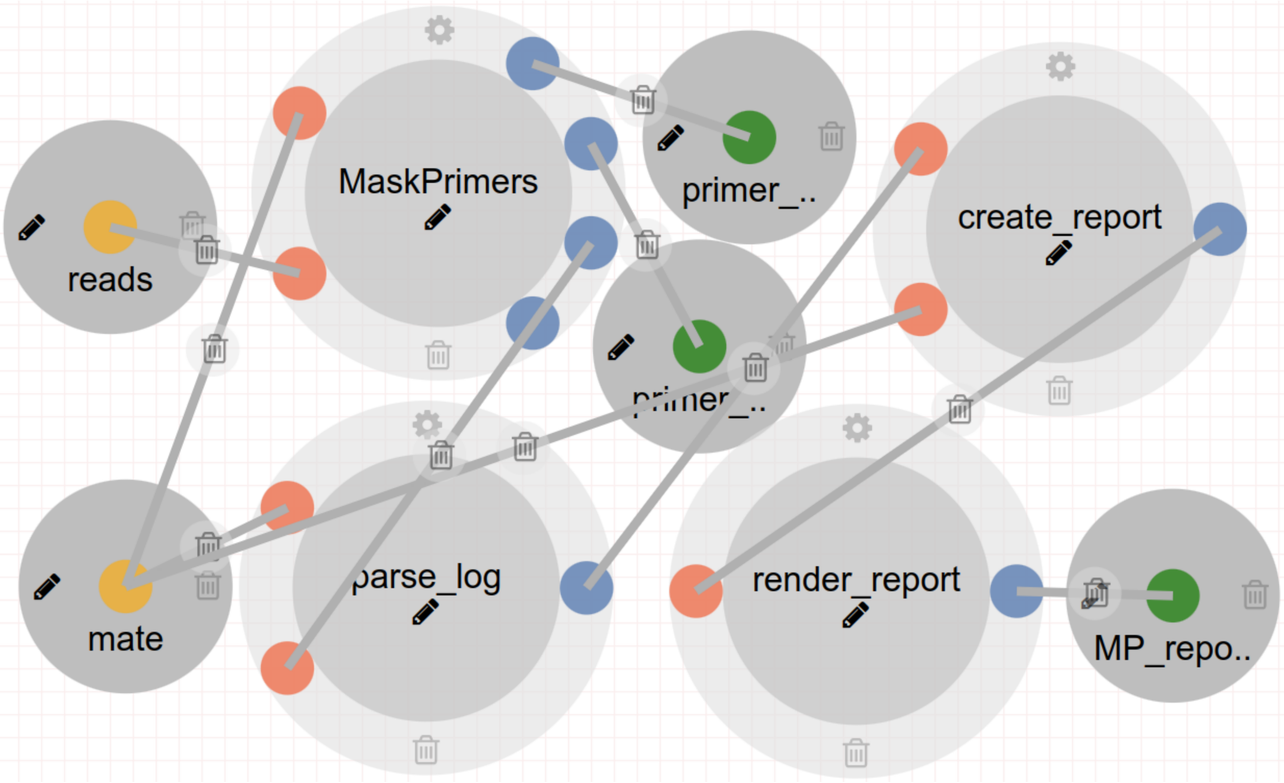

Figure S2: **ViaFoundry MaskPrimers module.** A screenshot of a ViaFoundry Module. The nodes are divided into three groups. The first, are small yellow nodes, these indicates input files or parameters. The second, are small green nodes, these indicated the output files from the process. The last group are the large gray nodes that indicated the process which the files are sent to. The lines connecting the nodes shows the transition of the files and parameters. The module shown in the figure shows the flow between the input to the MaskPrimer process to the log parsing process.

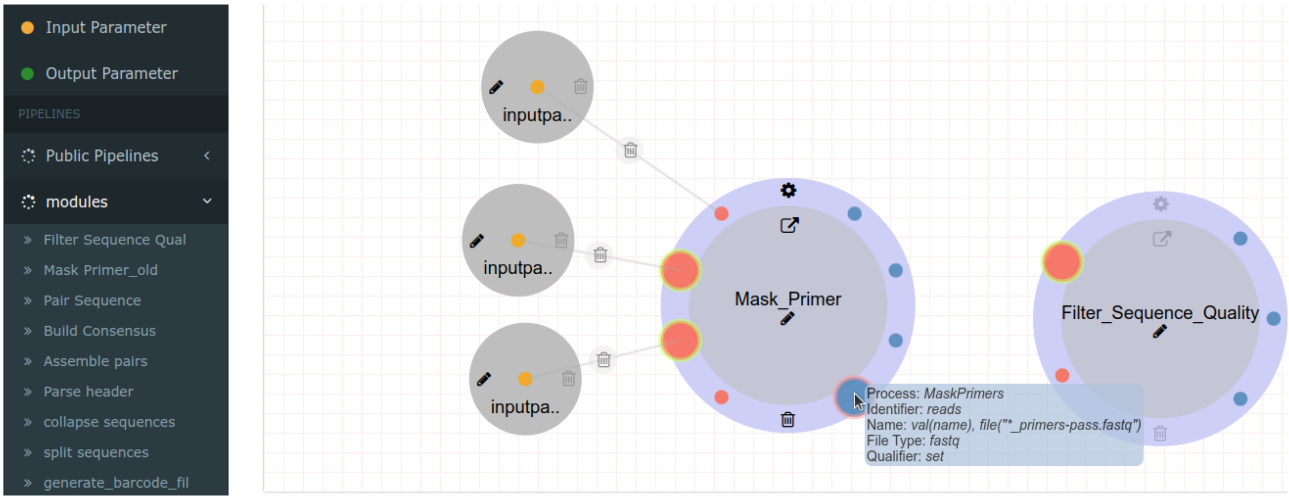

Figure S3: **ViaFoundry pipeline creation.** The screenshot shows the user interface of ViaFoundry to create a modular pipeline. The menu on the left shows the available input and output nodes, and the available models. The scheme on the right shows the connection between the two process. First we add the initial process, in this case Mask Primer, and then we connect the output of this process to the input of the following one (Filter sequence quality).

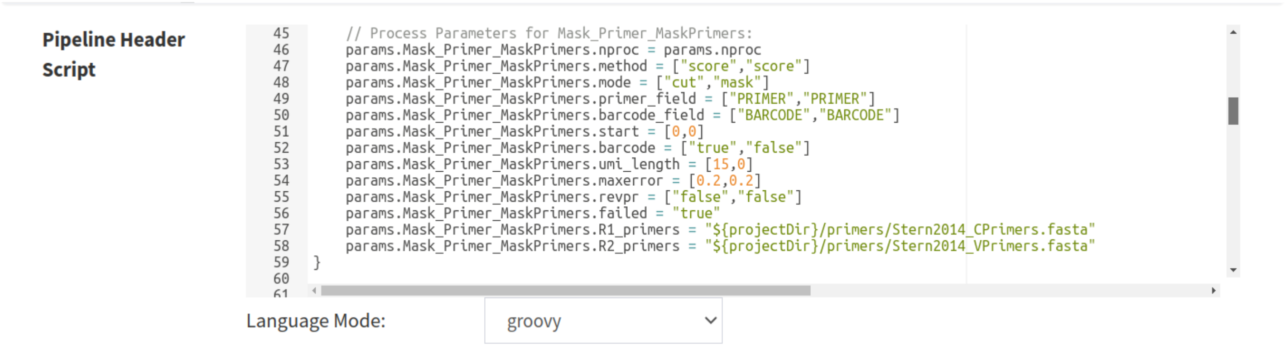

Figure S4: **Pipeline header parameter configuration.** The screenshot shows the section of the *Pipeline Header Script*. In this section, the user is given the capability to introduce extra inputs, scripts, comments, or fixate the parameters before the pipeline initiates. Lines 45-58 shows how we fixated the parameters for the MaskPrimers process for a specific pipeline.

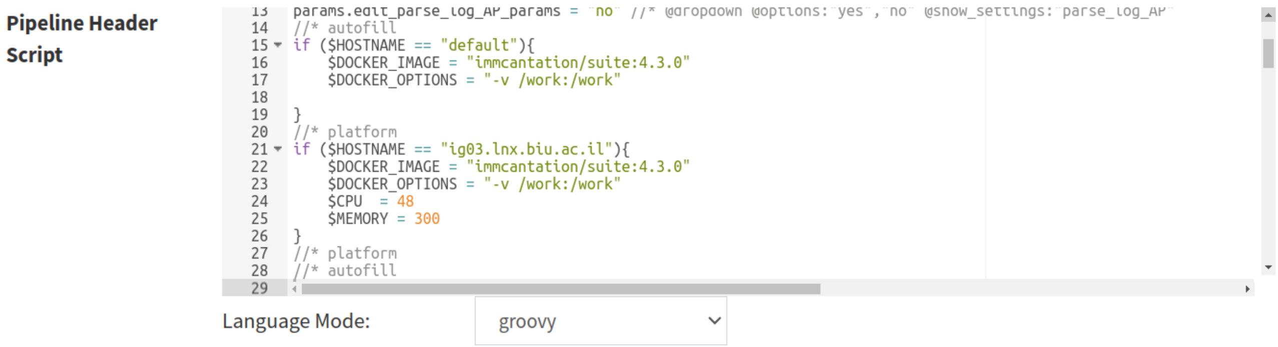

Figure S5: **Pipeline header run environment configuration.** The screenshot shows the section how to set the run environment in the *Pipeline Header Script*. Line 15 to 27 shows the setting of the run environment for different execution platforms.

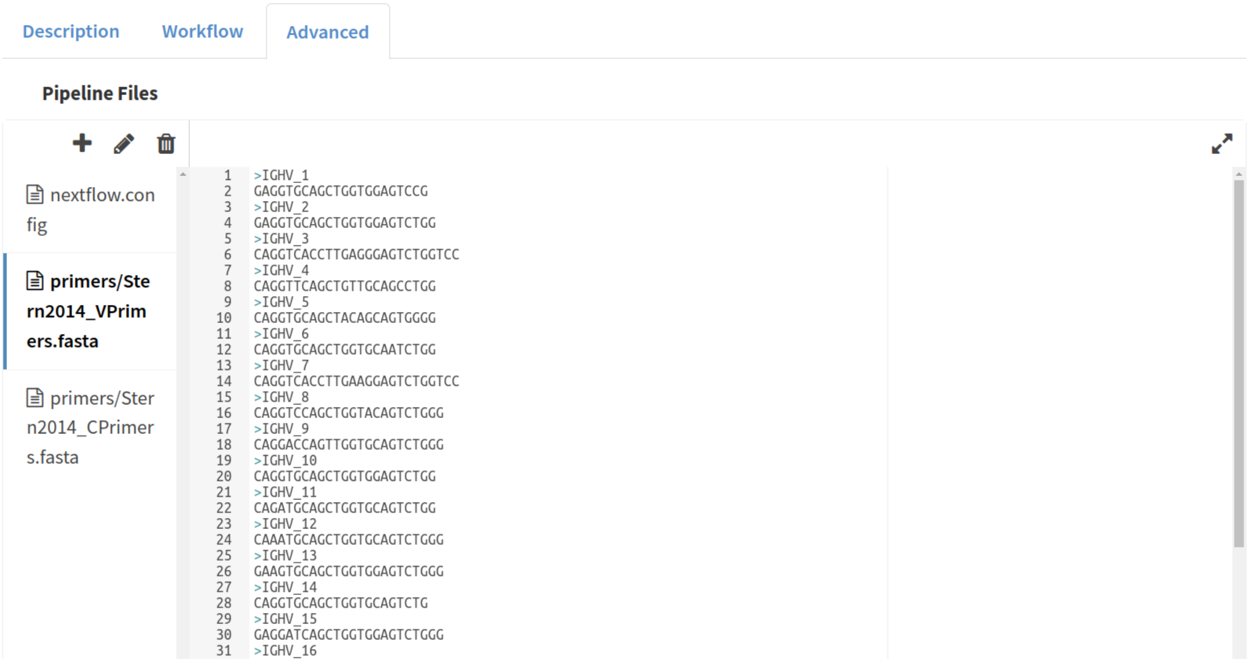

Figure S6: **Pipeline additional files.** The screenshot shows the *Pipeline Files* section that allows the user to add any additional files needed for executing the pipeline. For example, here the primer files used in the library preparation protocol for the AIRR-seq data were added. Once any file is added, it will be exported with the *Pipe.dn* file.
